# From Breath to Strength: Does Mindfulness Improve Handgrip Strength Among Older Adults in India? A Propensity Score Matching Analysis

**DOI:** 10.1111/psyg.70057

**Published:** 2025-06-22

**Authors:** T. Muhammad, Manacy Pai, Waad K. Ali, Boadi Agyekum, Shobhit Srivastava

**Affiliations:** ^1^ Center for Healthy Aging The Pennsylvania State University University Park Pennsylvania USA; ^2^ Department of Sociology and Criminology Kent State University Kent Ohio USA; ^3^ Department of Geography Sultan Qaboos University Muscat Oman; ^4^ School of Continuing and Distance Education, College of Education University of Ghana Accra Ghana; ^5^ Project Concern International Delhi India

**Keywords:** aging, handgrip strength, India, mindfulness activities

## Abstract

**Background:**

The integration of mindfulness activities into the daily lives of older adults has demonstrated profound benefits for their overall well‐being and vitality. However, evidence on how mindfulness correlates with muscle strength in older adults remains limited. To fill this gap, we explored the association between mindfulness activities and handgrip strength (HGS) in older adults in India. We also examined whether this association varies by sex.

**Methods:**

We employed a propensity score matching technique, leveraging data from the nationally representative Longitudinal Aging Study in India (2017–2018), comprising 27 071 adults aged 60 and above. HGS was measured using a handheld Smedley Hand Dynamometer while participants self‐reported their engagement in mindfulness activities.

**Results:**

Seventeen % of the men and 11.5% of the women engaged in mindfulness activities. Analysis of the matched sample revealed that the average treatment effect (ATE) that represents the average effect across the entire population was 1.28 kg for men and 0.60 kg for women, indicating that, on average, participation in mindfulness activities was associated with modest improvements in HGS for both sexes. Further, in the PSM matched regression models, for men, engagement in mindfulness activities was consistently associated with higher HGS across all models including the average treatment effect on the treated (ATT), on the untreated (ATU), and ATE, with the largest effect seen in the ATE model (*β* = 1.08, 95% CI: 0.77–1.40). For women, the association was weaker and significant only in the unmatched and ATE models.

**Conclusions:**

Engagement in mindfulness activities was associated with modest but meaningful improvements in HGS among older adults in India, with the associations being much more pronounced in older Indian men. These findings underscore the significance of integrating mindfulness practices into public health initiatives aimed at promoting healthier aging.

## 1 Background

As populations around the world continue to age, the promotion of healthy aging becomes increasingly imperative. Maintaining physical strength and vitality is crucial for older adults to preserve their independence, autonomy, and quality of life. The integration of mindfulness practices into the daily routines of older populations has been proven to improve their overall well‐being and vitality [1, 2]. Mindfulness activities such as meditation, yoga, and breathing exercises offer a range of mental, emotional, physical, and cognitive benefits to older individuals [3, 4, 5, 6, 7, 8]. However, limited research has explored the association between mindfulness practices and muscle strength, particularly among older adults in India.

HGS, a reliable marker of muscle strength and in turn, physical potency, is indicative of functional independence in older adults [9]. Extensive research underscores its pivotal role in older adults' well‐being—physical, mental, and musculoskeletal—making it an essential tool for gauging overall health and function [10]. Studies consistently link lower HGS to higher levels of depression among older adults [11, 12, 13, 14], patterns observed in Fukumori et al. across both cross‐sectional and longitudinal analyses [11, 15]. Furthermore, weak handgrip reliably indicates disability in older individuals, emphasizing its importance in assessing functional constraints and overall well‐being [16, 17, 18]. The significant association between physical inactivity and low HGS underscores its role in evaluating physical function and activity in older populations [19]. Moreover, HGS is a useful noninvasive indicator of musculoskeletal health [20, 21], with consequences for mobility, cognitive function, and early death. Therfore, interventions aimed at improving HGS can have significant implications for healthy aging.

Research on mindfulness has often focused on its advantages for pain management and mental wellness. For example, Morone et al. found that an eight‐session mindfulness course significantly reduced chronic pain in older participants in the intervention group compared with peers in the control group [22]. Similarly, Young and Baime found that mindfulness‐based stress reduction training reduced mental distress in 141 older participants, highlighting the role of mindfulness in improving emotional well‐being [4]. Furthermore, Geiger et al. reviewed the impact of mindfulness‐based interventions on the physical and emotional health of older persons [23], adding to the evidence that such practices support well‐being and physical function later in life [24, 25, 26, 27, 28].

Despite extensive work on mindfulness and its effects on mental health and pain, its impact on HGS, particularly in India, remains largely understudied. This gap is important because mindfulness has been linked to improved mental and physical function [1, 2], whereas HGS is a vital marker of overall health in aging populations [9, 10]. Given India's unique cultural and social context, it is important to explore how mindfulness may relate to muscle strength in this population. As such, we examined the association of mindfulness activities with HGS among older adults in India, employing a substantial, nationally representative sample and utilizing the propensity score matching methodology (PSM). We also evaluated whether this association varies between men and women, as the benefits of mindfulness for HGS may not be equally experienced by both sexes. We can gain a more thorough understanding of how mindfulness relates to physical health by examining the sex‐specific patterns in this relationship. It is important to note, however, that while PSM helps reduce selection bias, the cross‐sectional nature of the data still restricts our ability to make assertions regarding the relationship between mindfulness and HGS.

## 2 Methods

### 2.1 Data

Data were obtained from the baseline survey of the Longitudinal Aging Study in India (LASI), which was conducted between 2017 and 2018. The survey encompasses 72 250 individuals aged 45 years and older, representing all states and union territories of India on a national scale. The primary objective of the survey was to study the social, physical, mental, and cognitive health and well‐being of older Indians. The current analysis included respondents aged 60 years and over with a total sample of 27 071 older adults (men‐13 103 and women‐13 968).

LASI employes a complex sampling strategy utilizing a multistage stratified area probability cluster sampling design. This involves a three‐stage process in rural regions and a four‐stage process in urban areas. The detailed methodology, including specifics of the survey design and data collection, has been documented in earlier publications [29]. The data were anonymized, and all procedures adhered to relevant guidelines and regulations. Approval and ethical guidance for the LASI survey were secured from the Indian Council of Medical Research (ICMR).

### 2.2 Measures

#### 2.2.1 Outcome Variable (HGS)

Trained research assistants evaluated HGS in kilograms for both hands using a handheld Smedley Hand Dynamometer. The right forearm was positioned at the level of the elbow, with the upper arm held close to the torso. Participants were instructed to squeeze the dynamometer as firmly as possible for a brief period, three times with each hand. The highest value among the six attempts was recorded. The final HGS score (kg) was calculated as the average score (kg) of two successive trials in the dominant hand. We used the continuous measure of HGS for the analysis.

#### 2.2.2 Treatment Variable (Mindfulness Activities)

The mindfulness activities variable was constructed based on responses to the survey question: “How often do you engage in the activities such as yoga, meditation, asana, pranayama or similar?” The response options included (1). Every day (2). More than once a week (3). Once a week (4). One to three times a month (5). Hardly ever or never. For analysis, the variable was dichotomized as yes based on engagement in mindfulness activities at least once a month (responses 1–4, i.e., every day, more than once a week, once a week, one to three times a month) and no (response 5, i.e., hardly ever or never). In the sensitivity analysis, we classified those who engage in mindfulness activities at least once a week as yes and otherwise no (responses 4 and 5, i.e., one to three times a month, and hardly ever or never). Information on the frequency, duration, and specific type of practice was not available and therefore could not be assessed.

#### 2.2.3 Control Variables

Age was divided into three groups: of 60–69, 70–79, and 80+ years. Sex was coded as men or women. Education levels were classified as no education (including primary not completed), primary, secondary, and higher. Monthly per‐capita consumption expenditure (MPCE) quintiles were determined using household consumption data, divided into five quintiles, from poorest to richest, with detailed methodology outlined in the survey report. Marital status included currently married, widowed, and others. Others comprised those who were divorced/separated/never married. Religion was recoded as Hindu, Muslim, and others. Caste was recoded as Scheduled Caste/Scheduled Tribe (SC/ST), Other Backward Class (OBC), and others representing mainly those with higher social status. Place of residence was classified as urban versus rural. Regions were coded as North, Central, East, Northeast, West, and South.

We also included depression, body mass index, and number of chronic conditions as covariates. Depression was assessed using the Short Form Composite International Diagnostic Interview (CIDI‐SF) with a cut‐off score of 3 or more on a scale of 0–10 [30, 31]. Body mass index was assessed using the height and weight of participants, and classified as normal (18.5 to 24.9 $\mathrm{kg}/m^{2}$), underweight (less than 18.5 $\mathrm{kg}/m^{2}$), overweight (25–29.9 $\mathrm{kg}/m^{2}$) and obese (30 and above $\mathrm{kg}/m^{2}$), according to the WHO guidelines [32]. Number of chronic conditions was assessed using the self‐reporting of diagnosis by a health professional, and the conditions included hypertension, diabetes, heart attack, heart disease, stroke, lung disease, cancer, bone‐related disease, and neurological or psychiatric disorders including dementias. The variable was categorised into no disease, single, two, and three and more diseases.

### 2.3 Statistical Analysis

We employed propensity score matching (PSM) to analyze the treatment effects of mindfulness activities on HGS. PSM is a statistical method that minimizes selection bias, emulating an experimental design by ensuring compatibility in baseline characteristics and enabling the assessment of treatment effects in observational or cross‐sectional data. It creates a comparison group to address the counterfactual scenario, considering what the outcome would have been if the treatment had not been administered. In PSM, various observed predictors are used to create a propensity score, indicating the probability that each person is included in the treatment group. This score is then used to create a matched sample of treatment and control participants [33]. In this study, individuals engaged in mindfulness activities were assigned to the treatment group and matched with the control group members using a one‐to‐one matching method.

We performed multiple linear regression analyses in two stages: (1) pre‐matching, using the full sample to examine the association between mindfulness activities and HGS while adjusting for covariates, and (2) post‐matching, using the matched sample to refine the effect estimates. We also calculated the Average Treatment Effect on the Treated (ATT) and on the Untreated (ATU) to quantify the potential gain in HGS for participants and non‐participants, respectively, had they engaged in mindfulness activities [34]. For the PSM analysis, we utilised the “*psmatch2*” command in Stata (version 16) [35].

To assess covariate balance before and after matching, we report standardized mean differences, bias reduction percentages, and model diagnostics in Tables S1 and S2. These tables confirm that the matching procedure substantially improved balance between the treatment and control groups across covariates. To assess potential hidden bias from unobserved confounding, we also conducted a Mantel–Haenszel sensitivity analysis using the *mhbounds* command. A Gamma value of 1 suggests no evidence of hidden bias [36].

## 3 Results

**FIGURE 1:**
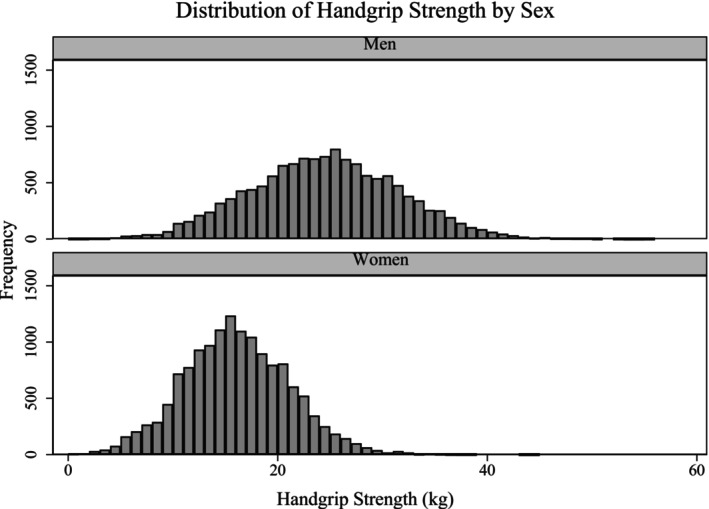
Histogram of handgrip strength (HGS) stratified by sex.

Table 1 represents the socio‐demographic and health profiles of older participants. 52.38% of the sample were females, 10.14% were in the age group of 80+ years, and more than half of the sample (56.44%) had no education, and 37.65% were not in a marital union at the time of the survey. Approximately 17% and 11.5% of older men and women were involved in mindfulness activities. Table 2 reveals the prevalence of engaging in mindfulness activities in older men, women, and the total sample, based on their background characteristics. A higher percentage of older men and women who were not educated/primary not completed, belonged to the poorest wealth quintile, were not in a marital union, resided in rural areas, and those who were underweight were not engaged in mindfulness activities. Figure 1 displays the histogram of HGS stratified by sex, showing a clear rightward shift in the distribution for older men compared to women, indicating that men generally exhibited higher HGS levels than their female counterparts.

**TABLE 1:** Socio‐demographic and health profile of older participants (*n* = 27 071).

Background variables	Men	Women	Total
	*n* (%)	*n* (%)	*n* (%)
Socio‐demographics			
Age (in year groups)			
60–69 years	7906 (59.49)	8764 (60.9)	16 670 (60.23)
70–79 years	3923 (30.13)	3884 (29.17)	7807 (29.63)
80+ years	1274 (10.38)	1320 (9.93)	2594 (10.14)
Sex			
Men			13 103 (47.62)
Women			13 968 (52.38)
Education			
No	4706 (38.53)	9742 (72.74)	14 448 (56.44)
Primary	2968 (22.78)	2142 (13.21)	5110 (17.77)
Secondary	3726 (25.95)	1572 (11.01)	5298 (18.12)
Higher	1703 (12.74)	512 (3.04)	2215 (7.66)
MPCE quintiles			
Poorest	2622 (20.93)	2908 (22.34)	5530 (21.67)
Poorer	2691 (21.63)	2925 (21.65)	5616 (21.64)
Middle	2680 (21.19)	2902 (20.55)	5582 (20.86)
Rich	2606 (19.49)	2734 (19.5)	5340 (19.49)
Richest	2504 (16.77)	2499 (15.96)	5003 (16.34)
Marital status			
In union	10 795 (81.15)	6585 (45.25)	17 380 (62.35)
Not in union	2308 (18.85)	7383 (54.75)	9691 (37.65)
Religion			
Hindu	9579 (82.44)	10 223 (82.72)	19 802 (82.59)
Muslim	1571 (11.15)	1628 (10.75)	3199 (10.94)
Others	1953 (6.41)	2117 (6.53)	4070 (6.47)
Caste			
SC	2142 (19.19)	2295 (18.89)	4437 (19.03)
ST	2151 (7.62)	2363 (8.53)	4514 (8.1)
OBC	5045 (45.83)	5249 (45.45)	10 294 (45.64)
Others	3765 (27.36)	4061 (27.13)	7826 (27.24)
Place of residence			
Urban	4222 (26.03)	4761 (30.14)	8983 (28.18)
Rural	8881 (73.97)	9207 (69.86)	18 088 (71.82)
Region			
North	2413 (12.54)	2590 (13.19)	5003 (12.88)
Central	1889 (22.88)	1796 (19.53)	3685 (21.12)
East	2579 (25.76)	2566 (23.5)	5145 (24.57)
Northeast	1576 (2.94)	1692 (2.99)	3268 (2.97)
South	2990 (20.12)	3365 (23.52)	6355 (21.9)
West	1656 (15.76)	1959 (17.27)	3615 (16.55)
Health‐related			
Depression			
No	12 299 (92.61)	12 920 (90.64)	25 219 (91.58)
Yes	804 (7.39)	1048 (9.36)	1852 (8.42)
Body mass index			
Normal	7345 (53.97)	6826 (48.43)	14 171 (51.07)
Underweight	3141 (28.44)	3182 (25.38)	6323 (26.84)
Overweight	2184 (14.8)	2808 (18.2)	4992 (16.58)
Obese	433 (2.78)	1152 (7.99)	1585 (5.51)
Chronic conditions			
None	6448 (50.4)	6174 (45.06)	12 622 (47.6)
Single	3698 (27.99)	4242 (30.16)	7940 (29.13)
Two	2003 (15.09)	2357 (15.53)	4360 (15.32)
Three and more	954 (6.52)	1195 (9.24)	2149 (7.95)
Mindfulness activities			
No	10 717 (82.94)	12 132 (88.5)	22 849 (85.85)
Yes	2386 (17.06)	1836 (11.5)	4222 (14.15)

**TABLE 2:** Prevalence of engaging in mindfulness activities among men, women, and total sample.

Background variables	Men (*n* = 2386, 17.06%)	Women (*n* = 1836, 11.50%)	Total sample (*n* = 4222, 14.15%)
	*n* (%)	*n* (%)	*n* (%)
Socio‐demographics			
Age (in year groups)			
60–69 years	1471 (17.58)	1249 (13.34)	2720 (15.33)
70–79 years	693 (16.22)	472 (9.44)	1165 (12.72)
80+ years	222 (16.52)	115 (6.31)	337 (11.28)
Education			
No	568 (10.32)	1029 (8.41)	1597 (9.03)
Primary	432 (14.27)	341 (16.24)	773 (15.04)
Secondary	784 (20.5)	308 (19.3)	1092 (20.12)
Higher	602 (35.4)	158 (36.57)	760 (35.64)
MPCE quintiles			
Poorest	273 (11.05)	222 (6.76)	495 (8.73)
Poorer	412 (14.44)	300 (9.56)	712 (11.88)
Middle	513 (17.29)	402 (12.37)	915 (14.75)
Rich	542 (19.93)	426 (13.04)	968 (16.32)
Richest	646 (24.3)	486 (17.78)	1132 (20.97)
Marital status			
In union	2010 (17.5)	1066 (14.93)	3076 (16.52)
Not in union	376 (15.14)	770 (8.67)	1146 (10.21)
Religion			
Hindu	1698 (16.36)	1230 (10.43)	2928 (13.25)
Muslim	219 (15.66)	162 (11.75)	381 (13.64)
Others	469 (28.48)	444 (24.69)	913 (26.48)
Caste			
SC	383 (15.22)	294 (9.83)	677 (12.42)
ST	222 (9.14)	213 (7.29)	435 (8.12)
OBC	857 (15.66)	584 (9.46)	1441 (12.42)
Others	924 (22.9)	745 (17.42)	1669 (20.04)
Place of residence			
Urban	980 (24.33)	808 (16.1)	1788 (19.72)
Rural	1406 (14.5)	1028 (9.52)	2434 (11.96)
Region			
North	750 (30.67)	676 (22.67)	1426 (26.38)
Central	301 (15.33)	172 (9.9)	473 (12.7)
East	549 (18.92)	411 (13.19)	960 (16.05)
Northeast	235 (17.99)	190 (11.71)	425 (14.67)
South	228 (6.95)	130 (3.92)	358 (5.24)
West	323 (18.41)	257 (12.78)	580 (15.33)
Health‐related			
Depression			
No	2212 (16.73)	1694 (11.54)	3906 (14.04)
Yes	174 (21.18)	142 (11.16)	316 (15.35)
Body mass index			
Normal	1330 (17.74)	841 (10.7)	2171 (14.24)
Underweight	392 (11.6)	259 (6.9)	651 (9.28)
Overweight	528 (22.44)	469 (16.7)	997 (19.14)
Obese	136 (30.93)	267 (19.16)	403 (21.99)
Chronic conditions			
None	1023 (14.16)	685 (9.04)	1708 (11.62)
Single	721 (18.67)	603 (12.93)	1324 (15.55)
Two	427 (20.76)	362 (15.8)	789 (18.13)
Three and more	215 (23.99)	186 (11.62)	401 (16.46)

Table 3 displays the estimates of the association between mindfulness activities and HGS among older men and women. The unmatched results revealed that, on average, HGS was higher among men and women who engaged in mindfulness activities in comparison to their peers who did not engage in mindfulness activities (difference: men, 2.12; women, 1.34). For the matched sample, among men, the ATT values were 26.11 for participants who engaged in mindfulness activities (treated group) and 23.99 for matched controls (non‐participants), indicating that engaging in mindfulness activities was associated with a 2.12 kg higher HGS. Among women, the corresponding ATT values were 17.15 for the treated group and 15.81 for controls, reflecting a 1.34 kg difference. The ATU values suggest that if non‐participants had engaged in mindfulness activities, their HGS would have increased from 25.03 to 26.11 (a 1.08 kg increase) among men, and from 16.96 to 17.15 (a 0.19 kg increase) among women. The ATE that represents the average effect across the entire population was 1.28 for men and 0.60 for women, indicating that, on average, participation in mindfulness activities was associated with modest improvements in HGS for both sexes.

**TABLE 3:** Results of matching estimates showing the effect of mindfulness activities on HGS among older adults.

Mindfulness activities	Treated	Control	Differences	SE	*T*‐stat
Men					
Unmatched	26.11	23.99	2.12	0.16	13.13
ATT	26.11	25.03	1.08	0.27	3.98
ATU	24.01	25.34	1.33		
ATE			1.28		
Women					
Unmatched	17.15	15.81	1.34	0.13	10.52
ATT	17.15	16.96	0.19	0.22	0.88
ATU	15.82	16.48	0.66		
ATE			0.6		
Total sample					
Unmatched	22.21	19.65	2.57	0.12	20.64
ATT	22.21	21.80	0.42	0.22	1.93
ATU	19.65	20.78	1.13		
ATE			1.02		

Abbreviations: ATT: Average Treatment Effect on the Treated; ATU: Average Treatment Effect on the Untreated; ATE: Average Treatment Effect (for the entire sample, regardless of treatment status).

In the total sample, unmatched results show that older adults who engaged in mindfulness activities had a 2.57 kg higher HGS than non‐participants. After matching, the ATT was 0.42 kg (*p* = 0.05), indicating a modest but statistically marginal improvement in HGS among participants. The ATU was 1.13 kg, and the ATE was 1.02 kg, suggesting that, on average, engagement in mindfulness activities is associated with a meaningful gain in HGS. As a sensitivity analysis, we considered engagement in mindfulness activities at least once per week. The results remained consistent, as presented in Table S3.

Table 4 displays the adjusted regression coefficients for HGS for the unmatched and matched (PSM) samples. In the unmatched sample, upon controlling for all background characteristics, the results revealed that engaging in mindfulness activities was significantly associated with higher HGS. The results are similar for the matched sample. Specifically, for men, engagement in mindfulness activities was consistently associated with significantly higher HGS across all models, with the largest effect seen in the ATE model (*β* = 1.08, 95% CI: 0.77–1.40). For women, the association was weaker and only statistically significant in the unmatched and ATE models. In the total sample, mindfulness activities were significantly associated with higher HGS, with *β* = 0.71 (95% CI: 0.52–0.90) in the unmatched model, *β* = 0.85 (95% CI: 0.61–1.09) for PSM ATE, and *β* = 0.63 (95% CI: 0.33–0.93) for PSM ATT.

**TABLE 4:** Adjusted regression coefficients for HGS in unmatched and PSM models.

Outcome variable (HGS)	Unmatched	PSM	
		ATE	ATT
Men			
Mindfulness activities (Yes vs. no)	0.97*** (0.67–1.27)	1.08*** (0.77–1.40)	0.80*** (0.41–1.19)
Women			
Mindfulness activities (Yes vs. no)	0.41** (0.17–0.65)	0.52*** (0.26–0.78)	0.17 (−0.15–0.49)
Total sample			
Mindfulness activities (Yes vs. no)	0.71*** (0.52–0.90)	0.85*** (0.61–1.09)	0.63*** (0.33–0.93)

*Note:* *p < 0.05, **p < 0.01, ***p < 0.001. The models were controlled for all the selected covariates.

Abbreviations: ATE: Average Treatment Effect (for the entire sample, regardless of treatment status); ATT: Average Treatment Effect on the Treated.

**FIGURE 2:**
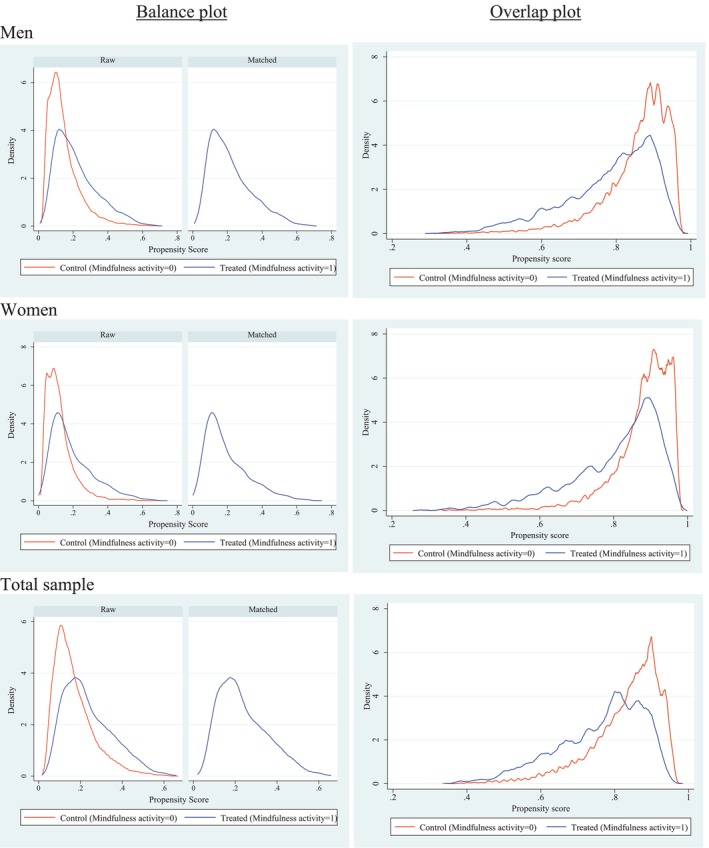
Balance plot before and after propensity matching, and overlap plot for propensity matching, for men, women, and total sample.

The kernel density plots of the propensity scores for the treatment and control groups, before and after matching, are shown in Figure 2. These plots illustrate that balance between control and treatment groups improved after matching. The overlap between distributions suggests that each participant had a positive probability of receiving treatment status, satisfying the overlap assumption. Minimal probability mass at the extremes (near 0 or 1) further supports this. Together, the evidence of covariate balance and adequate overlap indicates that the estimated treatment effects are likely unbiased, supporting the validity of the findings.

## 4 Discussion

The findings from the PSM analysis revealed important insights into the association between engagement in mindfulness activities and HGS, both before and after matching for various covariates. Prior to matching, our analysis indicated a noticeable discrepancy in HGS between older Indians who participated in mindfulness activities and those who did not. Specifically, the data revealed a notable average difference in HGS, with those engaged in mindfulness activities showing higher HGS levels than their counterparts. After conducting PSM to address potential confounding variables, the findings showed a nuanced yet positive linkage between mindfulness activities and HGS among older Indians. The ATT indicated a modest increase in HGS among older adults who engaged in mindfulness activities, even after considering covariate imbalances. Further, ATE indicated an improvement in HGS for the overall population if mindfulness activities were universally adopted. This finding suggests the potential public health benefits of promoting mindfulness practices among older Indians, with implications for enhancing physical strength and functional abilities.

Importantly, our results suggest that the positive association between mindfulness and HGS is much stronger and steadier among men. Older Indian men who practice mindfulness had considerably higher HGS than women across all models—ATT, ATU, and ATE—with the strongest association observed in the ATE model. Alternatively, the association for women was noticeably weaker. Moreover, among women, the association was statistically meaningful only in the unmatched and ATE models. This implies that although mindfulness may be beneficial to all older adults, a quantifiable difference in muscle strength only emerges among men. It would be worthwhile for those replicating this study to investigate the possibility that this discrepancy results from a mix of biological, behavioral, and environmental variations between men and women.

Interestingly, our analysis also explored the ATU, highlighting the potential gains in HGS among individuals not currently engaged in mindfulness activities if they were to initiate such practices. This finding underscores the relevance of mindfulness interventions as a means of enhancing physical well‐being among older adults, even among those not currently involved in such activities. Moreover, our adjusted regression analyses, both before and after PSM, consistently revealed the significant association between mindfulness activities and improved HGS, even after accounting for a range of sociodemographic and health factors.

These findings resonate with prior studies highlighting the positive impact of mindfulness activities, such as deep breathing, mindful meditation, music, and yoga [37, 38, 39], on HGS in older adults [20, 40, 41, 42, 43]. Notably, a meta‐analysis focusing on yoga's effects on physical fitness underscored its role in improving muscle strength, endurance, flexibility, balance, and coordination across various age groups [40]. The benefits of increased HGS include a lower risk of conditions like rheumatoid arthritis and diabetes [41] and better cognitive function in older adults [20]. Specifically, individuals with higher baseline HGS tend to achieve higher cognitive scores in the future [20]. Moreover, our study adds to the growing body of evidence suggesting a complex interplay between HGS and cardiovascular health in older adults. Previous research has identified positive correlations between HGS, blood pressure, and hypertension risk, highlighting the potential significance of HGS as a marker for cardiovascular health [42].

### 4.1 Study Limitations

*First*, although we relied on PSM to reduce selection bias and more closely mirror a quasi‐experimental design, the study remains cross‐sectional. As such, we cannot make inferences about cause and effect or the sequence of events. Future research, using forthcoming waves of LASI, can help assess whether sustained engagement in mindfulness leads to improvements in HGS over time. *Second*, while HGS is frequently utilized as a means of assessing muscle strength, future studies should consider incorporating other measures of muscle function, such as the chair rise test. *Third*, our study was limited to engagement in mindfulness activities. Examining activity types (e.g., meditation, yoga, breathing exercises), duration, and intensity may help understand the dose–response relationship, which is important in determining the optimal “dose” of mindfulness practice needed for meaningful improvements in HGS. Moreover, different individuals may respond differently to mindfulness practices. Some may benefit more from shorter, more intense sessions, while others may require longer, more moderate practice sessions to see improvements. Future surveys should collect detailed information to identify which practices are most effective.

## 5 Conclusion

Our study found an association between mindfulness activities and increased HGS in older persons in India, with stronger and more consistent benefits to older Indian men. For women, the association was noticeably weaker and inconsistent. That said, we observe a notable improvement in HGS among participants who exercise mindfulness relative to those who do not. Moreover, our analysis indicates a potential for even greater improvements in HGS for individuals not initially involved in mindfulness activities, should they choose to participate in them. These findings emphasize the value of integrating mindfulness techniques into public health initiatives aimed at promoting healthy aging. Such practices can help boost both mental and physical well‐being, stay active longer, and reduce health problems over time.

## Ethics Statement

Ethics approval was obtained from the Central Ethics Committee on Human Research (CECHR) under the Indian Council of Medical Research (ICMR) and the Institutional Review Boards of collaborating organisations including the International Institute for Population Sciences (IIPS), Mumbai, and the Ministry of Health and Family Welfare, Government of India. All methods were carried out in accordance with the relevant guidelines and regulations of ICMR.

## Consent

The survey agencies that conducted the field survey for the data collection have collected prior informed consent (signed and oral) for both the interviews and biomarker tests from the eligible respondents in accordance with Human Subjects Protection.

## Conflicts of Interest

The authors declare no conflicts of interest.

## Supporting information


**Data S1.** Supporting Information.

## Data Availability

The study uses secondary data that is available at the Gateway to Global Aging Data (https://g2aging.org/).
