# Supplementary material for: From Breath to Strength: Does Mindfulness Improve Handgrip Strength Among Older Adults in India? A Propensity Score Matching Analysis
Source: Psychogeriatrics. 2025 Jun 22;25(4):e70057. doi: 10.1111/psyg.70057 (PMC12182966; doi:10.1111/psyg.70057)
Supplement: Supplementary file 1 — Data S1. Supporting Information. [file PSYG-25-0-s001.docx]

**Supplementary materials**

**Table S1: Covariate balance statistics before and after PSM**

| Variables | Unmatched (U) vs Matched (M) | Mean | | %bias | %reduct bias | t-test | |
| --- | --- | --- | --- | --- | --- | --- | --- |
|  |  | Treated | Control |  |  | t | p>t |
| Age 2 | U | 0.276 | 0.291 | -3.3 |  | -1.94 | 0.052 |
|  | M | 0.276 | 0.272 | 0.9 | 71.1 | 0.44 | 0.66 |
| Age 3 | U | 0.08 | 0.099 | -6.7 |  | -3.85 | <0.001 |
|  | M | 0.08 | 0.078 | 0.5 | 92.5 | 0.24 | 0.809 |
| Sex 2 | U | 0.435 | 0.531 | -19.3 |  | -11.51 | <0.001 |
|  | M | 0.435 | 0.422 | 2.6 | 86.4 | 1.21 | 0.227 |
| Education 2 | U | 0.183 | 0.19 | -1.7 |  | -1.03 | 0.305 |
|  | M | 0.183 | 0.18 | 0.9 | 50.7 | 0.4 | 0.693 |
| Education 3 | U | 0.259 | 0.184 | 18 |  | 11.25 | <0.001 |
|  | M | 0.259 | 0.263 | -1 | 94.6 | -0.42 | 0.673 |
| Education 4 | U | 0.18 | 0.064 | 36.1 |  | 25.64 | <0.001 |
|  | M | 0.18 | 0.185 | -1.6 | 95.5 | -0.62 | 0.536 |
| Marital 2 | U | 0.271 | 0.374 | -22.1 |  | -12.81 | <0.001 |
|  | M | 0.271 | 0.245 | 5.7 | 74.4 | 2.76 | 0.006 |
| Marital 3 | U | 0.253 | 0.347 | -20.7 |  | -11.98 | <0.001 |
|  | M | 0.253 | 0.232 | 4.5 | 78.1 | 2.21 | 0.027 |
| Religion 2 | U | 0.09 | 0.123 | -10.7 |  | -6.12 | <0.001 |
|  | M | 0.09 | 0.12 | -9.7 | 9.8 | -4.47 | <0.001 |
| Religion 3 | U | 0.216 | 0.138 | 20.6 |  | 13.08 | <0.001 |
|  | M | 0.216 | 0.176 | 10.6 | 48.4 | 4.66 | <0.001 |
| Caste 2 | U | 0.341 | 0.387 | -9.6 |  | -5.68 | <0.001 |
|  | M | 0.341 | 0.34 | 0.3 | 96.9 | 0.14 | 0.89 |
| Caste 3 | U | 0.395 | 0.269 | 27 |  | 16.66 | <0.001 |
|  | M | 0.395 | 0.396 | -0.3 | 99.1 | -0.11 | 0.911 |
| Residence 2 | U | 0.577 | 0.685 | -22.6 |  | -13.82 | <0.001 |
|  | M | 0.577 | 0.558 | 3.8 | 83.2 | 1.69 | 0.091 |
| Region 2 | U | 0.112 | 0.141 | -8.6 |  | -4.97 | <0.001 |
|  | M | 0.112 | 0.131 | -5.8 | 32.8 | -2.7 | 0.007 |
| Region 3 | U | 0.227 | 0.183 | 11 |  | 6.73 | <0.001 |
|  | M | 0.227 | 0.222 | 1.3 | 88.2 | 0.57 | 0.566 |
| Region 4 | U | 0.101 | 0.124 | -7.5 |  | -4.36 | <0.001 |
|  | M | 0.101 | 0.108 | -2.5 | 67.1 | -1.17 | 0.241 |
| Region 5 | U | 0.085 | 0.262 | -48.2 |  | -25.32 | <0.001 |
|  | M | 0.085 | 0.113 | -7.8 | 83.9 | -4.41 | <0.001 |
| Region 6 | U | 0.137 | 0.133 | 1.3 |  | 0.8 | 0.425 |
|  | M | 0.137 | 0.109 | 8.2 | -519.9 | 3.94 | <0.001 |
| Diseases 2 | U | 0.314 | 0.29 | 5.2 |  | 3.15 | 0.002 |
|  | M | 0.314 | 0.323 | -2 | 62.6 | -0.89 | 0.375 |
| Diseases 3 | U | 0.187 | 0.156 | 8.1 |  | 4.97 | <0.001 |
|  | M | 0.187 | 0.184 | 0.8 | 90.7 | 0.34 | 0.737 |
| Diseases 4 | U | 0.095 | 0.076 | 6.6 |  | 4.08 | <0.001 |
|  | M | 0.095 | 0.089 | 2 | 69.2 | 0.9 | 0.367 |
| Depression 2 | U | 0.075 | 0.067 | 3 |  | 1.8 | 0.071 |
|  | M | 0.075 | 0.068 | 2.6 | 13 | 1.18 | 0.237 |
| BMI 2 | U | 0.154 | 0.248 | -23.6 |  | -13.31 | <0.001 |
|  | M | 0.154 | 0.171 | -4.3 | 81.6 | -2.15 | 0.031 |
| BMI 3 | U | 0.236 | 0.175 | 15.2 |  | 9.45 | <0.001 |
|  | M | 0.236 | 0.226 | 2.5 | 83.4 | 1.11 | 0.267 |
| BMI 4 | U | 0.095 | 0.052 | 16.8 |  | 11.14 | <0.001 |
|  | M | 0.095 | 0.079 | 6.3 | 62.6 | 2.66 | 0.008 |

**Table S2: Summary of the balance statistics before and after PSM**

| Sample | Ps R2 | LR chi2 | p>chi2 | Mean Bias | Med Bias | B | R |
| --- | --- | --- | --- | --- | --- | --- | --- |
| Unmatched | 0.113 | 2642.39 | <0.001 | 14.8 | 10.8 | 89.2* | 1 |
| Matched | 0.01 | 117.75 | <0.001 | 3.6 | 2.5 | 23.7 | 1.09 |
| * if B>25%, R outside [0.5; 2] | |  |  |  |  |  |  |

| **Table S3 Results of matching estimates showing the effect of mindfulness activities (at least once a week) on HGS among older adults** | | | | | |
| --- | --- | --- | --- | --- | --- |
| Mindfulness activities | Treated | Control | Differences | SE | T-stat |
| Men |  |  |  |  |  |
| Unmatched | 26.06 | 24.04 | 2.02 | 0.17 | 12.03 |
| ATT | 26.06 | 25.12 | 0.94 | 0.28 | 3.39 |
| ATU | 24.06 | 25.36 | 1.30 | . | . |
| ATE |  |  | 1.24 | . | . |
| Women |  |  |  |  |  |
| Unmatched | 17.09 | 15.84 | 1.25 | 0.13 | 9.42 |
| ATT | 17.09 | 16.75 | 0.34 | 0.22 | 1.55 |
| ATU | 15.84 | 16.48 | 0.64 | . |  |
| ATE |  |  | 0.61 | . |  |
| Total Sample |  |  |  |  |  |
| Unmatched | 22.18 | 19.70 | 2.48 | 0.13 | 19.17 |
| ATT | 22.18 | 21.80 | 0.38 | 0.22 | 1.72 |
| ATU | 19.70 | 20.62 | 0.92 | . | . |
| ATE |  |  | 0.84 | . | . |
| *The models were controlled for all the selected covariates* | | | | | |
